# Association between Occupational and Radiological Factors and Nontuberculous Mycobacteria Lung Infection in Workers with Prior Dust Exposure

**DOI:** 10.3390/ijerph16111966

**Published:** 2019-06-03

**Authors:** Ji-Won Lee, Jun-Pyo Myong

**Affiliations:** 1 Department of Research for Occupational Health, Institute of Occupation and Environment, Incheon 21417, Korea; celest2120@gmail.com; 2 Department of Occupational and Environmental Medicine, Seoul St. Mary’s Hospital, College of Medicine, The Catholic University of Korea, Seoul 06591, Korea

**Keywords:** nontuberculous mycobacteria, occupational exposure, pneumoconiosis

## Abstract

This retrospective cross-sectional study was conducted to identify the factors that promote the risk of nontuberculous mycobacteria (NTM) lung infection in subjects with prior occupational dust exposure. All consecutive patients with a history of occupational dust exposure whose expectorated sputum, bronchial wash, or bronchial lavage was subjected to acid-fast Bacilli culture in a tertiary hospital between 2011 and 2016 were identified. The patients who were infected with NTM were identified according to the bacteriological criteria of the American Thoracic Society (ATS) and The Infectious Diseases Society of America (IDSA) statement. Pneumoconiosis-associated radiological findings were graded according to the International Labor Organization guidelines. Of the 1392 patients with prior dust exposure, NTM was isolated from 82. Logistic regression analysis showed that risk factors for NTM lung infection were a history of pulmonary tuberculosis (adjusted odds ratio [aOR] = 1.82, 95% confidence intervals [CI] = 1.03–3.16). Moreover, the unadjusted odds ratios (ORs) were higher when both small-opacity profusion and the large-opacity grades increased. Even after adjustment, the ORs for the A, B, and C large-opacity grades were 2.32 (95% CI = 1.01–4.99), 2.68 (95% CI = 1.35–5.24), and 7.58 (95% CI = 3.02–17.95). Previous tuberculosis, bronchiectasis, and especially extensive small-opacity profusion, and high large-opacity grade associated significantly with NTM lung infection in dust-exposed workers.

## 1. Introduction

The nontuberculous mycobacteria (NTM) consist of all mycobacteria species except for *Mycobacterium tuberculosis* complex and *Mycobacterium leprae*. The most frequent target organ in NTM infections is the lung; more than 90% of NTM infections are in the lung [1,2]. Since untreated NTM lung disease can progress into extensive lung parenchymal destruction and death within years [1], there is an increasing interest in the diagnosis and treatment of NTM lung disease.

Risk factors for NTM lung disease include pulmonary conditions that associate with structural lung changes. These conditions include chronic obstructive pulmonary disease (COPD), bronchiectasis, previous tuberculosis (TB), and pneumoconiosis [3,4]. The latter disease, pneumoconiosis, is a restrictive lung disease that is caused by working for prolonged periods in dusty conditions (e.g., mining). Indeed, the Korean government considers NTM lung disease to be a complication of pneumoconiosis that deserves financial compensation.

While a history of silica exposure is a well-known risk factor for mycobacterial pulmonary infection in general [5], only a few clinical studies have addressed NTM infection in dust-exposed workers [6,7,8,9,10,11]. Moreover, none of these studies assessed the relationship between NTM infection and large opacities, which are a characteristic radiological feature of pneumoconiosis along with small opacities.

This retrospective cross-sectional study was conducted to identify the risk factors for NTM lung infection in patients who had a history of occupational dust exposure.

## 2. Methods

### 2.1. Patient Selection

This was a retrospective cross-sectional study that was conducted in a single hospital. The study cohort consisted of all consecutive inpatients and outpatients who had a history of occupational dust exposure and whose expectorated sputum, bronchial wash, or bronchial lavage was subjected to acid-fast Bacilli (AFB) culture in the Department of Occupational and Environmental Medicine of Seoul St. Mary’s Hospital between 1 May 2011 and 28 February 2016. Patients with respiratory symptoms were recruited, but those with respiratory symptoms and other diseases such as stroke and musculoskeletal diseases were excluded. If the patient had a history of exposure to cotton dust or asbestos, which causes diseases other than coal worker’s pneumoconiosis and silicosis, the patient was also excluded.

The study patients were considered to have an NTM lung infection on the basis of the diagnostic criteria for NTM lung disease that were established in 2007 by the American Thoracic Society (ATS) and The Infectious Diseases Society of America (IDSA) [3]. All subjects who had a dust-exposure history also had respiratory symptoms; therefore, it was hard to make a differential diagnosis based on the clinical criteria alone. Therefore, bacteriologic criteria alone were used to justify the case, and the term “NTM lung infection” was used instead of “NTM lung disease”. This study was reviewed and approved by the Institutional Review Board of Seoul St. Mary’s Hospital (KC17RESI0713).

### 2.2. Extraction of the General and Occupational Characteristics of the Patients

The age, medical history, and smoking status of each subject were extracted from the medical records. The patients were classified as a non-smoker (smoked ≤100 cigarettes in their lifetime and then stopped), ex-smoker (smoked >100 cigarettes in their lifetime and then quit), or current smoker (smoked >100 cigarettes in their lifetime and were currently smoking). The occupational characteristics, namely, type of job, duration of work, and type of dust to which the subjects had been exposed, were also determined. The type of job was classified as coal worker’s pneumoconiosis or another job involving dust exposure. If the patients had several jobs, the job that had the longest duration was selected.

### 2.3. Radiological Findings

All patients underwent standard chest radiographs around the time of their AFB culture. These radiographs were assessed for the characteristic radiological findings of pneumoconiosis, and the opacities were classified according to their concentration into the four profusion categories (0, 1, 2, or 3) of the International Labor Organization (ILO) classification system. ‘0’ profusion means that small opacities are absent, ‘1’ profusion indicates the presence of a few small opacities, ‘2’ profusion indicates the presence of partially obliterating vascular markings, and ‘3’ profusion signifies the presence of totally obliterating vascular markings. In addition, the large opacities (i.e., those whose longest dimension exceeded 10 mm) were graded as A, B, or C opacities on the basis of the ILO classification system. ‘A’ indicates one (or more) large opacities whose (combined) longest dimension is 10–50 mm. ‘B’ indicates one (or more) large opacities whose (combined) longest dimension exceeds 50 mm but does not exceed the equivalent area of the right upper lung zone. ‘C’ indicates one (or more) large opacities whose (combined) longest dimension exceeds the equivalent area of the right upper lung zone [12].

Whether the patients had pulmonary comorbid radiological findings, namely, pneumoconiosis with emphysema, bronchitis, bronchiectasis, or cavitary lesion, was also assessed. These assessments were preferentially made on the basis of computed tomography (CT) chest images around the time that the AFB cultures were being performed. However, if the subjects did not undergo chest CT, the chest x-rays were used to assess comorbidity with emphysema, bronchitis, and bronchiectasis. The presence of a cavitary lesion was confirmed on the basis of chest CT only because some records did not mention the existence of a cavity.

### 2.4. Statistical Analysis

The patients with and without NTM were compared in terms of demographic, radiological, and clinical variables. Wilcoxon rank sum test was used to compare the two groups in terms of age and work duration because these continuous variables did not have normal distributions. The Chi-squared test and Fisher’s exact test were used to compare the two groups. Multiple logistic regression analysis was performed to identify risk factors for NTM. Adjustments were made for age, work duration, smoking status, small-opacity profusion category, large-opacity grade, presence of bronchiectasis, and presence of a pulmonary cavity. The data were expressed as Odds ratios (OR) and 95% confidence intervals (CI). *p*-values of <0.05 were considered to indicate statistical significance. All analyses were performed using the Statistical Analysis System (SAS) version 9.4 (SAS Institute, Cary, NC, USA).

## 3. Results

In total, 1438 patients underwent an AFB culture of their sputum, bronchial wash, or lavage sample(s) during the study period. Of these, 46 were excluded because they had other diseases or were exposed to other dust such as asbestos. Therefore, the study cohort consisted of 1392 patients.

Of the 1392 subjects, 82 received a microbiological diagnosis of pulmonary NTM infection. The mean age of all subjects was 67.8 ± 8.1 years, and the mean duration of dusty work was 21.7 ± 10.7 years. Compared with the patients without NTM lung infection, the patients with NTM lung infection were significantly more likely to be in their 60 s and 70 s, to have diabetes and hypertension, to have a history of pulmonary TB, and to have been a coal worker. In terms of radiological features, the patients with lung NTM infection were significantly more likely than the patients without this infection to have category 2 and 3 small-opacity profusion and higher large-opacity grades (Table 1). They were also significantly more likely to have bronchiectasis and especially emphysema (Table 2).

Multiple logistic regression analysis showed that, before adjustment, a history of pulmonary TB (OR = 2.29, 95% CI = 1.34–3.84) and the presence of bronchiectasis (OR = 1.90, 95% CI = 1.00–3.38) were risk factors for NTM lung infection (Table 3). Moreover, the risk of NTM lung infection rose with the degree of small-opacity profusion: category 1, 2, and 3 small-opacity profusion associated with crude ORs of 1.48 (95% CI = 0.68–3.47), 3.48 (95% CI = 1.75–7.72), and 4.02 (95% CI = 1.62–10.24), respectively. The risk of NTM lung infection also rose as the large-opacity grade rose: grades A, B, and C associated with crude ORs of 2.92 (95% CI = 1.56–5.27), 2.74 (95% CI = 1.53–4.81), and 7.15 (95% CI = 3.15–15.11), respectively.

When adjustments were made for age, work duration, history of pulmonary TB, bronchiectasis, pulmonary cavity, and small-opacity profusion (Model 1), history of pulmonary TB (OR = 1.82, 95% CI = 1.03–3.16) and category 2 (OR = 2.79, 95% CI = 1.25–7.10), and 3 (OR = 3.76, 95% CI = 1.36–10.94) small-opacity profusion remained risk factors for NTM lung infection. When the same adjustments were made except that small-opacity profusion was substituted by the large-opacity grade (Model 2), grade A (OR = 2.32, 95% CI = 1.01–4.99), B (OR = 2.68, 95% CI = 1.35–5.24), and C (OR = 7.58, 95% CI = 3.02–17.95), large opacities were risk factors for NTM lung infection.

The NTM species in 43 of 82 NTM lung infected patients were identified. A total of 61 outcomes were identified, including the isolation of multiple species from the same patient. Identified NTM species are shown in Table 4. Some species from respiratory specimens were considered not pathogenic and to be contaminants. *M. gordonae* in seven patients and *M. terrae* complex in one patient were also considered contaminants.

## 4. Discussion

Immunocompromised patients and patients with a pre-existing pulmonary disease often develop NTM infections, of which the *Mycobacterium avium* complex (MAC) are the most common agents. This study showed that the risk factors for a diagnosis of NTM lung infection in patients with prior occupational dust exposure were previous TB, bronchiectasis, extensive small-opacity profusion, and higher large-opacity grades. Even after adjustment, extensive small-opacity profusion and higher grade large opacities remained significant risk factors for NTM lung infection in dust-exposed workers.

While prior TB infection was a significant risk factor for NTM lung infection in the unadjusted model and Model 1, it had an increased OR without statistical significance in Model 2. This finding is consistent with the case-control study of Corbett et al., who showed that prior TB infection was also a risk factor for NTM lung disease in South African gold miners [13]. Corbett et al. suggested a post-tuberculous structure distortion and poor immunologic response to Mycobacterium antigen after TB infection to explain the association between prior TB and susceptibility to NTM lung disease. It is also largely accepted that bronchiectasis associates with NTM lung disease [14,15,16,17]. However, the statistical significance of the association was not observed after adjusting for other factors such as the type of dust exposure and pneumoconiosis. Pneumoconiosis, the immune reaction induced by macrophages that remove deposited dust in the lung, and the epithelial fibrosis induced by inflammatory cells, such as polymorphonuclear leukocytes (PMN), are likely to cause structural changes [18]. All patients in this study had pneumoconiosis, which distorts lung structure as a result of the immunologic reaction to inhaled dust. Since patients are vulnerable to NTM pulmonary infection, the history of tuberculosis may have lost its significance after adjustment for the profusion of small opacity perfusion or large opacity grades. Also, prior TB is merely a marker of the poor ability of the individual to respond immunologically to mycobacterial antigens.

Although the results of univariate analyses showed that emphysema also associated significantly with NTM lung disease, emphysema was excluded from the analysis because it correlated with large-opacity grade (Data not shown). Note that, because the large opacities, which indicate progressive massive fibrosis (PMF) caused by pneumoconiosis, are commonly accompanied by paracicatricial emphysema [19], which can be seen next to areas of scarring, emphysema was not included in the multivariate analysis.

In addition, Corbett et al. found that a dust-exposed job at diagnosis was a risk factor for NTM lung disease in the South African gold miners, even after adjustment for silicosis [13]. This is consistent with the study of Sonnenberg et al., which showed that the miners who had spent more than 20 years underground were more likely to have NTM lung disease than those who had spent less than 10 years underground [6]. Silica exposure may promote NTM lung disease via two mechanisms. First, people who work in a dusty environment may have a higher risk of TB lung disease and chronic bronchitis, both of which promote susceptibility to NTM lung disease [6,20]. This possibility is consistent with the study by Beamer et al. on mice that were instilled intranasally with silica. They found that silica exposure altered the phenotype of the alveolar macrophages, which in turn impaired their ability to take up and respond protectively to bacterial lipoproteins [21]. Second, not only is dust a common source of NTM, wet processes in association with a dusty job environment have the potential to increase the incidence of pathogenic mycobacterial exposure in the workplace [13,22,23].

Corbett et al. also reported that the gold miners with NTM were significantly more likely to have early/high-grade silicosis than the control patients, who were gold miners who attended hospital for trauma or human immunodeficiency virus (HIV)-unrelated surgery [13]. However, it was not clear how they graded silicosis. Consequently, whether their early/high-grade silicosis category reflects the higher ILO grades of large opacities in complicated pneumoconiosis or only greater small profusion opacities in simple pneumoconiosis is unclear. However, another, more recent, study by Sonnenberg et al. reported that South African gold miners with NTM lung disease were significantly more likely than gold miners with TB to exhibit silicosis, as defined by ILO categories of the small-opacity profusion of 1/1 or higher. This relationship between NTM lung disease and silicosis remained significant even after adjusting for age, alcohol consumption, HIV status, years spent working underground, prior TB treatment, and silicosis [10]. Similarly, we found that NTM-infected patients with previous dust exposure were significantly more likely than uninfected dust-exposed patients to have category 2 and 3 small-opacity profusion. These findings suggest that pneumoconiosis promotes NTM lung disease.

To the best of our knowledge, the association between large-opacity grade (as shown by chest X-rays) and the risk of NTM lung infection has not been studied. We found that patients with NTM lung infection were significantly more likely to have large-opacity grade A, B, and C radiological findings and that the association between severe large opacity and NTM lung infection remained significant after adjustment. Alternatively, it may be that large opacities lead to lung distortion, which could cause ventilatory disturbances. Emphysema is the result of lung parenchymal destruction and is a type of COPD that restricts airflow. Ringshausen et al. showed that diagnosed COPD (either the chronic bronchitis-predominant type or the emphysema-predominant type) associated with NTM lung disease [24]. Chan et al. suggested that the low blood supply of emphysematous lesions encourages the colonization of NTMs in these areas [25].

This study has several limitations. First, its cross-sectional design made it difficult to assess the causal relationship between NTM lung infection and radiological factors. However, given the natural course of pneumoconiosis and the fact that the radiological charts were obtained around the time NTM was isolated, it is likely that there is a causal relationship, namely, that the structural lung changes in pneumoconiosis promote NTM lung infection. Second, the subjects were enrolled in a single center, which may reduce the generalizability of the study observations to other settings. However, this limitation may be mitigated by the fact that the subjects worked in heterogeneous workplaces and varied in their occupational history. Third, we did not analyze the pulmonary function test data. Further studies on the relationship between NTM lung infection and lung function are warranted. Fourth, the NTM species could not be identified in many cases; as a result, we could not assess their relationship with NTM lung infection in pneumoconiosis. However, all NTM lung infections were confirmed according to the American Thoracic Society (ATS) and The Infectious Diseases Society of America (IDSA) guidelines. Fifth, some of the subjects who had decreased FEV1 (Forced expiratory volume in one second) might have used inhaled corticosteroids, and most patients under medication might have used acid-suppressive drugs as part of a clinical regimen. Unfortunately, it was difficult to evaluate the medication history data in the analysis because of the retrospective design of the study. Additionally, although slender body habitus has been reported to be associated with NTM, infections, we did not evaluate individual anthropometric data such as height, weight, body mass index, mainly because the main focus of our study was the association between radiologic findings of pneumoconiosis and NTM lung infection. Considering that some of them needed medical treatment including inhaled corticosteroids to relieve their respiratory symptoms and most patients with a history of dust exposure who complain of chronic respiratory symptoms have slender body habitus, the present study suggests that occupational history of respiratory dust and its complex health effect are a risk factor for NTM pulmonary infection.

Despite these limitations, this study also has a number of methodologically robust elements. First, the NTM-infected patients satisfied the bacteriological criteria of the ATS/IDSA statement about NTM diagnosis. Second, there were 82 patients with NTM lung infection, which is markedly larger than the sample sizes in other studies on NTM in pneumoconiosis. Third, the occupational history data included the work duration. Consequently, we could examine the relationship between dust exposure duration and NTM infection. Fourth, this is the first study that evaluates the relationship between the large opacities of pneumoconiosis and NTM lung infection.

## 5. Conclusions

The diagnosis of NTM lung infection in dust-exposed workers was significantly associated with previous TB, bronchiectasis, marked profusion of small opacities, and high-grade large opacities, as assessed using the ILO classification system. Even after adjustment, extensive small-opacity profusion and high-grade large opacities remained significant risk factors for NTM lung infection in dust-exposed workers. The relationship between pneumoconiosis features and NTM species, and the relationship between lung function and NTM lung infection remain to be studied.

## Figures and Tables

**Table 1:** Differences between patients with and without NTM lung infection in terms of demographic, occupational, and radiological characteristics.

Total		No Recovery of Lung NTM, *n* (%)	Recovery of Lung NTM, *n* (%)	*p*-Value ^a^
		1310 (94.1)	82 (5.9)	
Age, years				
	40–49	11 (0.8)	3 (3.6)	0.0052 *
	50–59	216 (16.5)	4 (4.9)	
	60–69	495 (37.8)	33 (40.3)	
	70–79	513 (39.2)	39 (47.6)	
	≥80	75 (5.7)	3 (3.6)	
Work duration, years				
	<10	273 (20.8)	7 (8.5)	0.0550
	10–19	354 (27.0)	30 (36.6)	
	20–29	338 (25.8)	25 (30.5)	
	30–39	238 (18.2)	13 (15.9)	
	≥40	107 (8.2)	7 (8.5)	
Gender				
	Male	1258 (96.0)	82 (100)	0.0659
	Female	52 (4.0)	0 (0)	
Diabetes ^†^				
	No	749 (78.8)	39 (72.2)	0.2495
	Yes	201 (21.2)	15 (27.8)	
Hypertension ^‡^				
	No	552 (56.2)	30 (50)	0. 3514
	Yes	431 (43.8)	30 (50)	
History of pulmonary tuberculosis ^§^				
	No	905 (77.7)	38 (60.3)	0.0015 *
	Yes	260 (22.3)	25 (39.7)	
Smoking status				
	Non-smoker	191 (21.3)	17 (22.4)	0.0644
	Ex-smoker	571 (63.7)	55 (72.4)	
	Current smoker	134 (15.0)	4 (5.2)	
Type of job				
	Coal worker	932 (71.2)	68 (82.9)	0.0214 *
	Other	378 (28.9)	14 (17.1)	
Pneumoconiosis characteristics				
Profusion of small opacities				
	0	319 (24.4)	9 (11.0)	<0.0001 *
	1	456 (34.8)	19 (23.2)	
	2	438 (33.4)	43 (52.4)	
	3	97 (7.4)	11 (13.4)	
Grade of large opacities				
	No	926 (70.7)	35 (42.7)	<0.0001 *
	A	154 (11.8)	17 (20.7)	
	B	193 (14.7)	20 (24.4)	
	C	37 (2.8)	10 (12.2)	

^a^*p*-values were obtained by comparing the groups using the Chi-squared test or Fisher’s exact test. * Statistically significant values (*p* < 0.05). ^†^ 388 missing data on diabetes. A total of 1004 subjects were included. ^‡^ 349 missing data on hypertension. A total of 1043 subjects were included. ^§^ 164 missing data on tuberculosis. A total of 1228 subjects were included. 420 missing data on smoking status. A total of 972 subjects were included. NTM, nontuberculous mycobacteria.

**Table 2:** Differences between patients with and without NTM lung infection in terms of selected radiological features.

		No Recovery of Lung NTM, *n* (%)	Recovery of Lung NTM, *n* (%)	*p*-Value ^a^
Pulmonary cavity on chest CT ^†^				0.1954
	No	764 (88.3)	65 (83.3)	
	Yes	101 (11.7)	13 (16.7)	
Bronchitis				1.0000
	No	1289 (98.4)	81 (98.8)	
	Yes	21 (1.6)	1 (1.2)	
Bronchiectasis				0.0341 *
	No	1182 (90.2)	68 (82.9)	
	Yes	128 (9.8)	14 (17.1)	
Emphysema				<0.0001 *
	No	811 (61.9)	22 (26.8)	
	Yes	499 (38.1)	60 (73.2)	

^a^*p*-values were obtained by comparing the groups using the Chi-squared test or Fisher’s exact test. * Statistically significant values (*p* < 0.05). ^†^ 449 missing data on pulmonary cavity on chest CT. A total of 943 subjects were included. CT, computed tomography; NTM, nontuberculous mycobacteria.

**Table 3:** Multiple logistic regression analysis of the relationship between nontuberculous mycobacteria infection and occupational, clinical, and radiological characteristics.

		Unadjusted Model		Model 1 ^†^		Model 2 ^‡^	
		OR	95% CI	OR	95% CI	OR	95% CI
Age (per year)		1.02	0.99–1.05	1.01	0.98–1.05	1.01	0.98–1.05
Work duration, years							
	<20	1.00		1.00		1.00	
	≥20	1.12	0.71–1.76	0.92	0.53–1.59	0.89	0.51–1.55
History of pulmonary tuberculosis							
	No	1.00		1.00		1.00	
	Yes	2.29 *	1.34–3.84	1.82 *	1.03–3.16	1.71	0.95–3.01
Bronchiectasis							
	No	1.00		1.00		1.00	
	Yes	1.90 *	1.00–3.38	0.99	0.43–2.03	1.01	0.44–2.09
Pulmonary cavity on chest CT							
	No	1.00		1.00		1.00	
	Yes	1.51	0.77–2.76	1.47	0.69–2.90	1.34	0.62–2.70
Profusion of small opacities							
	0	1.00		1.00			
	1	1.48	0.68–3.47	1.22	0.47–3.36		
	2	3.48 *	1.75–7.72	2.79 *	1.25–7.10		
	3	4.02 *	1.62–10.24	3.76 *	1.36–10.94		
Grade of large opacities							
	No	1.00				1.00	
	A	2.92	1.56–5.27			2.32 *	1.01–4.99
	B	2.74	1.53–4.81			2.68 *	1.35–5.24
	C	7.15 *	3.15–15.11			7.58 *	3.02–17.95

* Statistically significant values (*p <* 0.05). ^†^ Model 1: Adjusted for age, work duration, history of pulmonary tuberculosis, bronchiectasis, pulmonary cavity, and profusion of small opacities. ^‡^ Model 2: Adjusted for age, work duration, history of pulmonary tuberculosis, bronchiectasis, pulmonary cavity, and grade of large opacities. OR, odds ratio; CI, confidence interval.

**Table 4:** NTM infected patients with species identification.

Species	Number of Patients	Number of Patients with Other Species	Other Species
*M. avium*	13	3	*M. kansasii, M. abscessus, M. fortuitum, M. gordonae*
*M. intracellulare*	13	5	*M. fortuitum, M. gordonae*
*M. fortuitum*	10	8	*M. avium, M. intracellulare, M. gordonae, M. abscessus, M. peregrinum, M. lentiflavum, M. celatum*
M. gordonae *	7	5	*M. avium, M. intracellulare, M. fortuitum, M. abscessus, M. smegmatis, M. lentiflavum*
*M. abscessus*	4	3	*M. avium, M. fortuitum, M. gordonae*
*M. peregrinum*	3	2	*M. fortuitum, M. lentiflavum*
*M. smegmatis*	3	2	*M. gordonae, M. lentiflavum*
*M. lentiflavum*	3	3	*M. fortuitum, M. gordonae, M. peregrinum, M. smegmatis*
*M. kansasii*	1	1	*M. avium*
*M. chelonae*	1	0	
*M. celatum*	1	0	
M. terrae complex *	1	0	
Other type	1	0	
Total	61		

* Species generally considered as environmental contamination. Three patients were identified with more than three species.

## References

[1] Koh W.-J. (2011). Diagnosis and treatment of nontuberculous mycobacterial lung disease. J. Korean Med. Assoc..

[2] Park Y.S., Lee C.H., Lee S.M., Yang S.C., Yoo C.G., Kim Y.W., Han S.K., Shim Y.S., Yim J.J. (2010). Rapid increase of non-tuberculous mycobacterial lung diseases at a tertiary referral hospital in South Korea. Int. J. Tuberc. Lung Dis..

[3] Griffith D.E., Aksamit T., Brown-Elliott B.A., Catanzaro A., Daley C., Gordin F., Holland S.M., Horsburgh R., Huitt G., Iademarco M.F. (2007). An official ATS/IDSA statement: Diagnosis, treatment, and prevention of nontuberculous mycobacterial diseases. Am. J. Respir. Crit. Care Med..

[4] Huang C.T., Tsai Y.J., Wu H.D., Wang J.Y., Yu C.J., Lee L.N., Yang P.C. (2012). Impact of non-tuberculous mycobacteria on pulmonary function decline in chronic obstructive pulmonary disease. Int. J. Tuberc. Lung Dis..

[5] Pasula R., Britigan B.E., Turner J., Martin W.J. (2009). Airway delivery of silica increases susceptibility to mycobacterial infection in mice: Potential role of repopulating macrophages. J. Immunol..

[6] Sonnenberg P., Murray J., Glynn J.R., Thomas R.G., Godfrey-Faussett P., Shearer S. (2000). Risk factors for pulmonary disease due to culture-positive *M. tuberculosis* or nontuberculous mycobacteria in South African gold miners. Eur. Respir. J..

[7] Kim Y.M., Kim M., Kim S.K., Park K., Jin S.H., Lee U.S., Kim Y., Chae G.T., Lee S.B. (2009). Mycobacterial infections in coal workers’ pneumoconiosis patients in South Korea. Scand. J. Infect. Dis..

[8] British Thoracic and Tuberculosis Association (1975). Opportunist mycobacterial pulmonary infection and occupational dust exposure: An investigation in England and Wales. Tubercle.

[9] Corbett E.L., Hay M., Churchyard G.J., Herselman P., Clayton T., Williams B.G., Hayes R., Mulder D., De Cock K.M. (1999). Mycobacterium kansasii and *M. scrofulaceum* isolates from HIV-negative South African gold miners: Incidence, clinical significance and radiology. Int. J. Tuberc. Lung Dis..

[10] Sonnenberg P., Thomas R.G., Glynn J.R., Shearer S., Godfrey-Faussett P., Murray J. (2005). Clinical and radiological features of pulmonary disease due to culture-positive *M. tuberculosis* or nontuberculous mycobacteria in South African gold miners. S. Afr. J. Epidemiol. Infect..

[11] Van Halsema C.L., Chihota V.N., Gey van Pittius N.C., Fielding K.L., Lewis J.J., van Helden P.D., Churchyard G.J., Grant A.D. (2015). Clinical Relevance of Nontuberculous Mycobacteria Isolated from Sputum in a Gold Mining Workforce in South Africa: An Observational, Clinical Study. Biomed. Res. Int..

[12] Jo B.S., Lee J., Cho Y., Byun J., Kim H.R., Koo J.W., Myong J.P. (2016). Risk factors associated with mortality from pneumonia among patients with pneumoconiosis. Ann. Occup. Environ. Med..

[13] Corbett E.L., Churchyard G.J., Clayton T., Herselman P., Williams B., Hayes R., Mulder D., De Cock K.M. (1999). Risk factors for pulmonary mycobacterial disease in South African gold miners. A case-control study. Am. J. Respir. Crit. Care Med..

[14] Griffith D.E., Aksamit T.R. (2012). Bronchiectasis and nontuberculous mycobacterial disease. Clin. Chest Med..

[15] McShane P.J., Glassroth J. (2015). Pulmonary Disease Due to Nontuberculous Mycobacteria: Current State and New Insights. Chest.

[16] Weiss C.H., Glassroth J. (2012). Pulmonary disease caused by nontuberculous mycobacteria. Expert Rev. Respir. Med..

[17] Aksamit T.R., Philley J.V., Griffith D.E. (2014). Nontuberculous mycobacterial (NTM) lung disease: The top ten essentials. Respir. Med..

[18] Rom W.N. (2015). Environmental and Occupational Medicine.

[19] Cockcroft A.E., Wagner J.C., Seal E.M., Lyons J.P., Campbell M.J. (1982). Irregular opacities in coalworkers’ pneumoconiosis—Correlation with pulmonary function and pathology. Ann. Occup. Hyg..

[20] Hnizdo E., Murray J. (1998). Risk of pulmonary tuberculosis relative to silicosis and exposure to silica dust in South African gold miners. Occup. Environ. Med..

[21] Beamer G.L., Seaver B.P., Jessop F., Shepherd D.M., Beamer C.A. (2016). Acute Exposure to Crystalline Silica Reduces Macrophage Activation in Response to Bacterial Lipoproteins. Front. Immunol..

[22] Primm T.P., Lucero C.A., Falkinham J.O. (2004). Health impacts of environmental mycobacteria. Clin. Microbiol. Rev..

[23] Ulmann V., Kracalikova A., Dziedzinska R. (2015). Mycobacteria in Water Used for Personal Hygiene in Heavy Industry and Collieries: A Potential Risk for Employees. Int. J. Environ. Res. Public Health.

[24] Ringshausen F.C., Wagner D., de Roux A., Diel R., Hohmann D., Hickstein L., Welte T., Rademacher J. (2016). Prevalence of Nontuberculous Mycobacterial Pulmonary Disease, Germany, 2009–2014. Emerg. Infect. Dis..

[25] Chan E.D., Iseman M.D. (2013). Underlying host risk factors for nontuberculous mycobacterial lung disease. Semin. Respir. Crit. Care Med..

